# Integrative rare disease biomedical profile based network supporting drug repurposing or repositioning, a case study of glioblastoma

**DOI:** 10.1186/s13023-023-02876-2

**Published:** 2023-09-25

**Authors:** Erin McGowan, Jaleal Sanjak, Ewy A. Mathé, Qian Zhu

**Affiliations:** Division of Pre-Clinical Innovation National Center for Advancing Translational Sciences (NCATS), National Institutes of Health (NIH), 9800 Medical Center Drive, Rockville, MD 20850 USA

**Keywords:** Data integration, Network analysis, Drug repurposing or repositioning, Glioblastoma

## Abstract

**Background:**

Glioblastoma (GBM) is the most aggressive and common malignant primary brain tumor; however, treatment remains a significant challenge. This study aims to identify drug repurposing or repositioning candidates for GBM by developing an integrative rare disease profile network containing heterogeneous types of biomedical data.

**Methods:**

We developed a Glioblastoma-based Biomedical Profile Network (GBPN) by extracting and integrating biomedical information pertinent to GBM-related diseases from the NCATS GARD Knowledge Graph (NGKG). We further clustered the GBPN based on modularity classes which resulted in multiple focused subgraphs, named mc_GBPN. We then identified high-influence nodes by performing network analysis over the mc_GBPN and validated those nodes that could be potential drug repurposing or repositioning candidates for GBM.

**Results:**

We developed the GBPN with 1,466 nodes and 107,423 edges and consequently the mc_GBPN with forty-one modularity classes. A list of the ten most influential nodes were identified from the mc_GBPN. These notably include Riluzole, stem cell therapy, cannabidiol, and VK-0214, with proven evidence for treating GBM.

**Conclusion:**

Our GBM-targeted network analysis allowed us to effectively identify potential candidates for drug repurposing or repositioning. Further validation will be conducted by using other different types of biomedical and clinical data and biological experiments. The findings could lead to less invasive treatments for glioblastoma while significantly reducing research costs by shortening the drug development timeline. Furthermore, this workflow can be extended to other disease areas.

**Supplementary Information:**

The online version contains supplementary material available at 10.1186/s13023-023-02876-2.

## Background

Glioblastoma (GBM) is a rare, malignant variety of brain tumor that develops from astrocyte and oligodendrocyte cells. [1] GBM is both the most aggressive and most common malignant primary brain tumor, making up 54% of all gliomas and 16% of all primary brain tumors. [2] The incidence of GBM ranges from 0.59 to 5 per 100,000 persons, and this number is rising in many countries. [3] GBM patients have a median survival of only 15 months, and less than 5% of patients survive 5 years following diagnosis. There is currently no cure for GBM. [2] The standard of care (SOC) for grades 3 or 4 high-grade gliomas (HGGs) including GBM, a grade 4 glioma, currently relies on maximally safe surgical resection followed by concurrent radiation therapy and temozolomide (TMZ). [4] While this SOC has increased the median survival time of GBM patients, [2] Stupp et al. [5] has shown that the two year survival rate of patients who undergo radiotherapy plus TMZ treatment is only 26.5%. Moreover, Stupp et al. [5] found that concomitant treatment with radiotherapy plus TMZ resulted in grade 3 or 4 hematologic toxic effects in 7% of patients.

By April 2022, four drugs besides TMZ were approved by the FDA for treating HGGs: lomustine, [6] intravenous carmustine, [7] carmustine implants, [8] and bevacizumab, [9]. One device, tumor treatment fields (a cap containing electrodes which deliver alternating electric fields to a patient’s scalp that disrupt tumor growth), was also FDA-approved for the treatment of HGGs. [10] Only TMZ, carmustine implants, and tumor treatment fields are FDA-approved for new diagnoses (the rest are approved for recurrent HGGs). [4] Carmustine wafer implants are expensive and have a high complication rate (42.7%), whereas tumor treatment fields are expensive, inconvenient for patients, and yield marginal survival benefits. [4] Toxicity is also a common issue with all current therapies. [4] Thus, there is an urgent need for therapy discovery for GBM patients that are both effective and less invasive than the current SOC.

Drug repurposing is the practice of repurposing an active pharmaceutical ingredient already approved for use in the treatment of one condition for the treatment of another (we differentiate this from drug repositioning, which we will use to refer to the practice of finding a new use for drugs that had some other intended purpose in clinical trials, but do not have regulatory approval). [11] This approach reduces research costs and allows treatments to reach patients more quickly. Repurposed drugs seeking approval are 150% more likely to be introduced on the market than novel drugs. [12] The exponential growth of large-scale, publicly-available biomedical and pharmaceutical data combined with advancements in high-performance computing have enabled the development of various computational drug repurposing approaches including data mining, machine learning, and network analysis. [13] These in silico strategies, along with disease molecular profiles (e.g. associated genes, biomarkers, signaling pathways, environmental factors, etc.), empower researchers to determine the degree of similarity between diseases by their molecular features. [11] Network analysis in particular has been used extensively in computational drug repurposing, as networks provide an intuitive method of modeling biological and biomedical entities and their interactions and relationships to each other. [13] Centrality measures play a vital role in network analysis, allowing researchers to identify important nodes within a network from a structural perspective. [14] Though frequently used in social network analysis, centrality measures have been adapted as a metric for biological studies since as early as 2001. [15] A previous drug repurposing study ranked drugs by their centrality scores within networks composed of drugs connected based on their side effects and interactions. [16] Another study suggests that the centralities of drugs in a network of drugs connected based on their side-effect similarities may have significant implication in drug repurposing. [14] Most of those published applications mainly leveraged one aspect of drugs, such as side effects or interactions; thus nodes in their established network were specifically associated with drugs (as opposed to other data types such as diseases, phenotypes, proteins, etc.). Inspired by these studies, we proposed to generate integrative rare disease biomedical profiles with heterogenous types of data from our previously developed NCATS Genetic and Rare Diseases (GARD) Knowledge Graph (NGKG), [17] which contains information about diseases, genes, drugs, pathways, cells, etc. pooled from forty-three rare disease-related data resources, which can be found in the supplemental file named “NGKG Resources”. Furthermore, instead of mining the entire NGKG, only GBM-associated subgraphs based on pre-calculated disease clusters were derived, and multiple network analysis techniques, such as centrality measures and community detection, were combined and applied to generate GBM-focused graphs for identifying high-influence nodes, which might be potential drug repurposing or repositioning candidates.

## Materials and methods

**Fig. 1:**
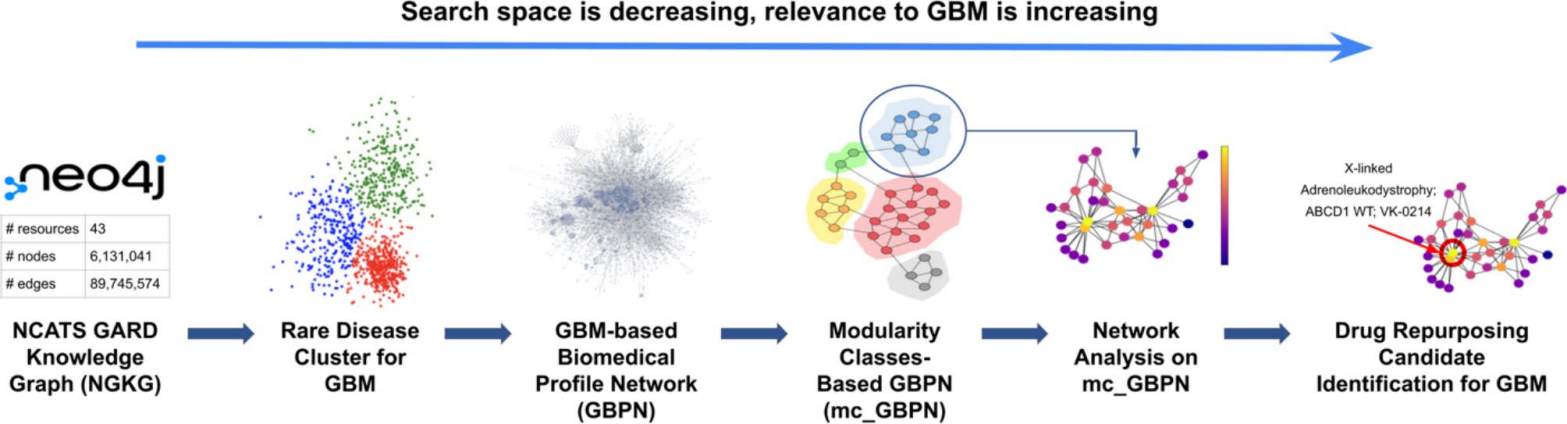
Workflow for identifying drug repurposing or repositioning candidates for GBM.

In this study, to uncover significant associations relevant to GBM for drug repurposing or repositioning, we performed network analysis in three steps: (1) we developed a GBM-based Biomedical Profile Network (GBPN) by obtaining the GBM-related biomedical data extracted from the NGKG, [17] (2) we clustered the GBPN into a modularity classes-based network (mc_GBPN) by performing community detection, and (3) we identified high-influence nodes as potential candidates for drug repurposing or repositioning for GBM from mc_GBPN via various centrality measures. In other words, these steps allow us to create a large graph (GBPN) containing all information related to GBM from the NGKG, group nodes containing similar information into broad categories (mc_GBPN), and then identify the most “important” nodes in each category, respectively. The “importance” of each node will be defined by an average of several metrics (see Section D), each of which identify nodes that are integral to the graph’s structure by different measures. Figure 1 shows the study workflow.

### NCATS gard knowledge graph (NGKG)[17

**Fig. 2:**
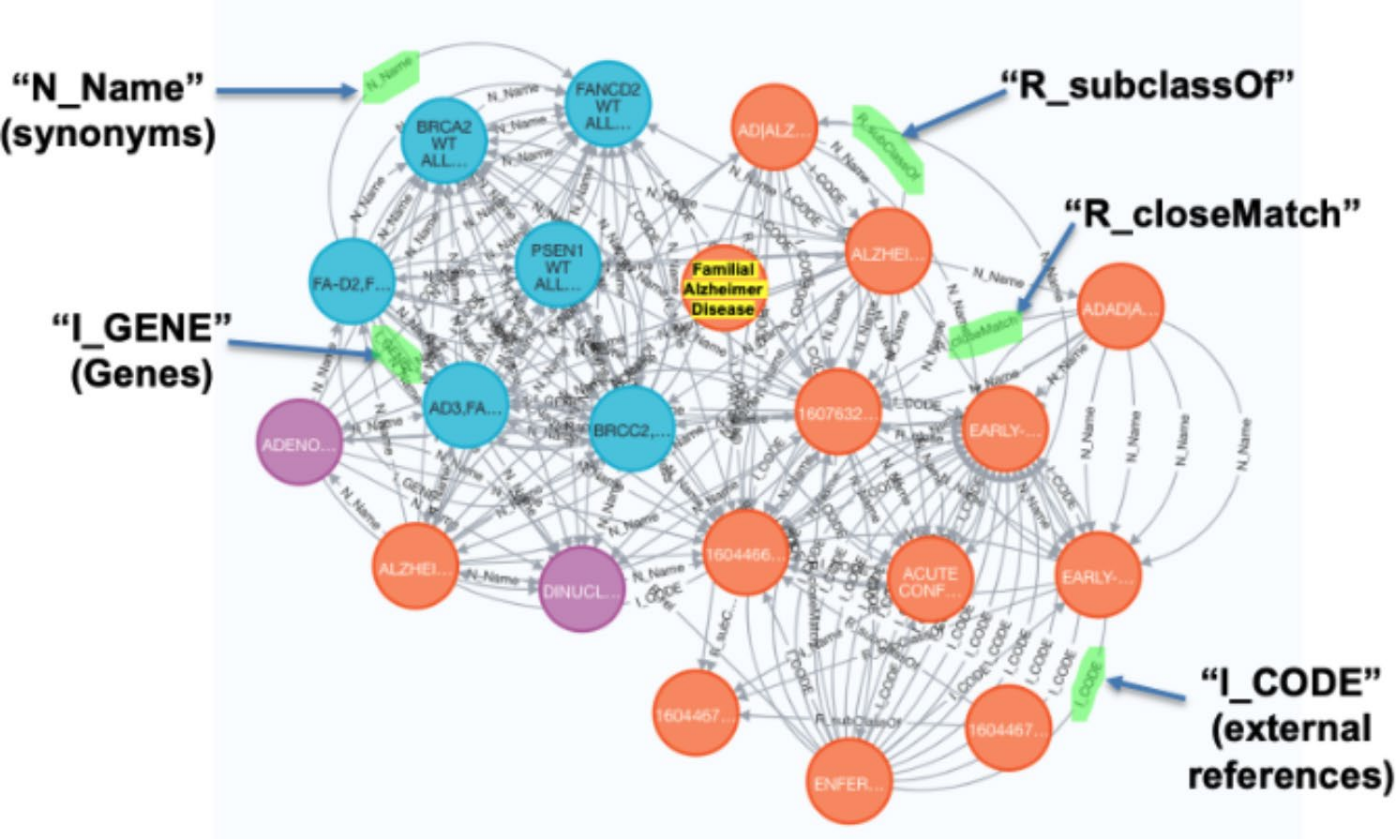
Familial Alzheimer Disease-based subgraph derived from the NGKG. Orange nodes denote diseases, blue nodes denote genes, and purple nodes denote drugs. Familial Alzheimer Disease is highlighted in yellow

The GARD Information Center was managed by the NCATS to provide freely accessible consumer health information on over 10,000 genetic and rare diseases. To expand the use of information from GARD for biomedical research in rare diseases, we previously developed the NGKG, [17] a knowledge graph that integrated data from GARD and other well-known rare disease related resources including Orphanet, [18] OMIM, [19] MONDO, [20] and curated mappings between FDA orphan designations to GARD, and information on FDA approval status and drug indications from Inxight Drugs, [17] using our stitcher [21] software. The full list of fourty-three resources in the supplemental file named “NGKG Resources”. Stitcher defines edges to link equivalent/relevant concepts from different resources; for instance, “N_Name” denotes linked concepts with the same concept names, while “I_CODE” denotes linked concepts sharing the same external reference. In addition, stitcher adopts predicates from original resources, such as “R_equivalentClass” from MONDO. More examples are shown in Fig. 2.

### GBM-based biomedical profile network (GBPN)

#### Development

To construct the GBPN with GBM-relevant information, we generated a disease cluster pertinent to GBM. This cluster containing GBM and 91 other GBM-related rare diseases was generated through a modified version of DL2Vec [22] applied to data obtained from the NGKG and enriched with additional data sources. Specifically, a focused subgraph of the NGKG was extracted containing disease, genes and phenotypes. The subgraph was annotated with Gene Ontology [23] and Human Phenotype Ontology [24] and then enriched with small molecule and pathway data from Pharos [25] and The Pathway Commons, [26] respectively. Random walks emanating from each rare disease were used to generate a corpus from which disease node embeddings were created. The disease node embeddings were clustered using the k-means algorithm. Detailed description of the disease clustering procedure has been described in a separate submission. [27].

We extracted 92 subgraphs from the NGKG, each an ego graph [28] of radius of 3 centered on a node containing one of those 92 GBM-related rare diseases. Figure 2 shows one subgraph that is centered on the node of Familial Alzheimer Disease, one disease from the GBM-related disease cluster. We then merged the union of these subgraphs to create the GBPN.

#### Optimization

**Fig. 3:**
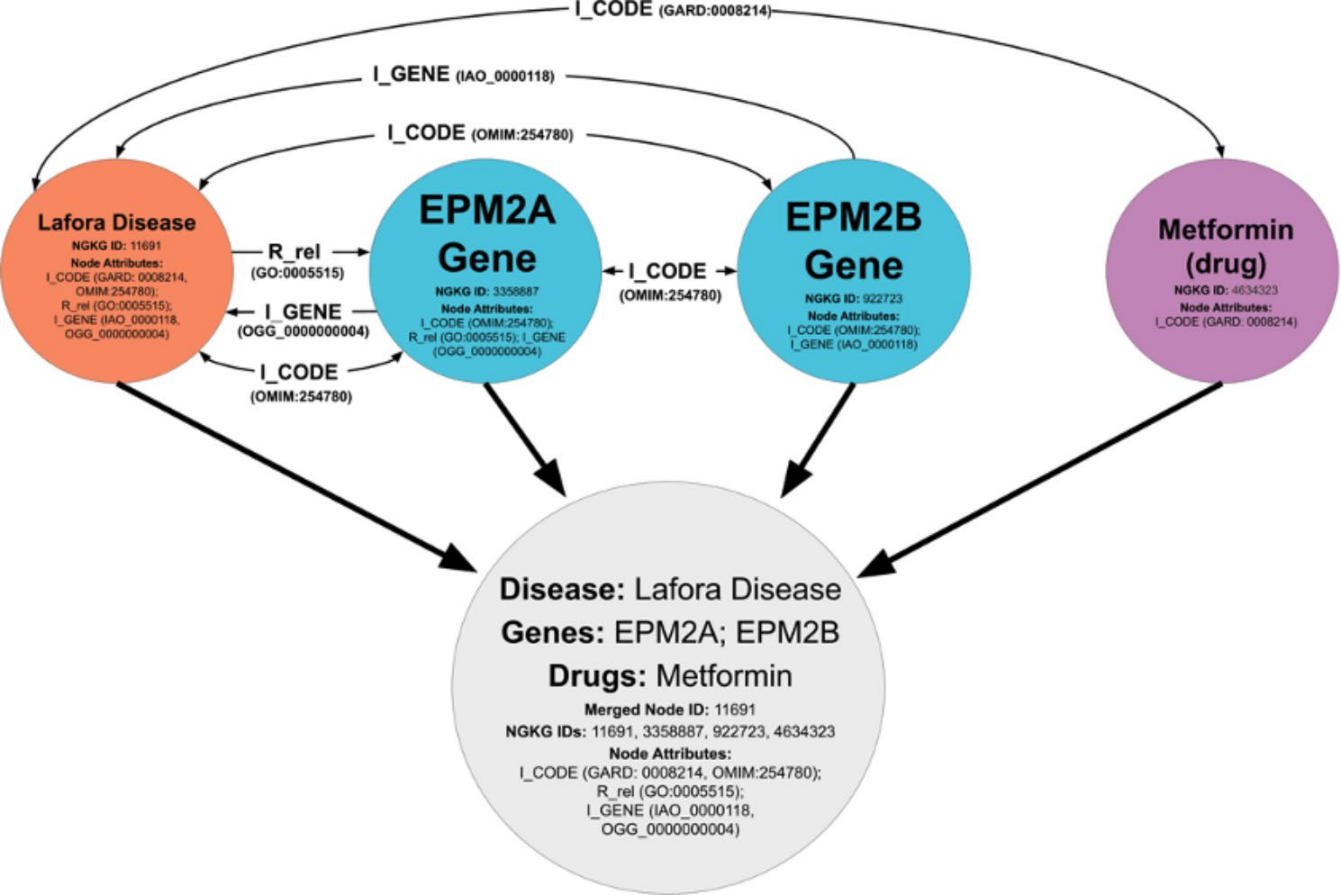
A node containing Lafora disease is merged with nodes connected to it by an edge label of “I_CODE”: two Lafora disease related genes, the EPM2A gene, the EPM2B gene, and Metformin, a treatment that has been used for Lafora disease. The gray node is one of the merged nodes in the GBPN.

The NGKG maintains connections among equivalent or relevant concepts from different resources via pre-defined edges, e.g. “N_Name” and “I_CODE” or those adopted predicates, e.g. “R_equivalentClass” and “R_exactMatch”. We optimized the GBPN by merging associated diseases, genes, treatments, etc. with those aforementioned edges into singular nodes, yielding a more condensed graph of nodes with enriched biomedical information for efficient network analysis. Specifically, we optimized the GBPN via these rules: 1) the attributes of merged nodes were concatenated; 2) edges were removed if the connected nodes were merged (i.e. if nodes A and B merged, all edges between A and B would be removed); 3) edges were maintained between unmerged and newly-merged nodes (i.e. if node A and B merged into node AB, an edge from A to node C would be reassigned as an edge from AB to C). The code used to implement rules 1–3 is in the supplemental materials. Synonyms were subsequently filtered out of name labels within newly merged nodes. For instance, if the nodes “Addison’s Disease” and “Adrenal aplasia’’ were merged, both of these labels (which denote the same disease) would be concatenated within the newly merged node. In this case, we would verify that “Adrenal aplasia” is a synonym of “Addison’s Disease” by querying the NGKG for the “synonyms” attribute of the “Addison’s Disease” node and would subsequently remove “Adrenal aplasia” from the newly merged node’s name label in the GBPN. This process was automated and applied to each newly merged node; some other complementary resources, including the NORD Rare Diseases database, [29] GeneCards, [30] the National Library of Medicine’s MedlinePlus, [31] PubChem, [32] and the National Cancer Institute’s List of Cancer Drugs, [33] were applied for this process as well. Figure 3 illustrates one merging example.

### mc_GBPN development

To group the GBPN into focused subgraphs, we clustered the GBPN into modularity classes (mc_GBPN) using community detection [34] available from Gephi 0.9.6. [35] Gephi is an open source tool for creating and exploring interactive network visualizations that includes functions for network analysis. Gephi uses the Louvain modularity algorithm [36] for community detection, which maximizes a modularity score for each community and is well-suited to large networks. [37] We set randomize to “On” and the resolution to 1.0. Smaller resolution values recover more communities (each containing fewer nodes), while larger resolution values recover fewer communities (each containing more nodes). [38] While larger resolution values may fail to separate distinct communities, [39] smaller resolution values may produce communities that are too small to perform meaningful network analysis on. In the case of the GBPN, resolution values less than 1.0 translated to over half of the communities being too small (three nodes or fewer) to analyze. We prioritized the mc_GBPN by modularity score and the top ten mc_GBPN were applied for further investigation. Specifically, we sorted mc_GBPN with more than three nodes in descending order by modularity score. The modularity score of a mc_GBPN is defined as $\frac{{{L_c}}}{m} - \gamma {(\frac{{{k_c}}}{{2m}})^2}$where ${L}_{c}$ is the number of intra-community edges for mc_GBPN, ${k}_{c}$ is the sum of degrees of the nodes in mc_GBPN, $m$ is the total number of edges across all mc_GBPN, and $\gamma$ is the resolution parameter (in this case, 1.0). [40–41] A mc_GBPN with a higher modularity score contains more internal-connections and less external-connections, which results in a large number of “hub nodes” with high centrality scores and therefore is of interest to our investigation for drug repurposing. Thus, we sought out mc_GBPN with a high modularity score.

The mc_GBPN were then reviewed and assigned a class label based on parent-child relationships denoted in the NGKG and Disease Ontology. [42] For example, one mc_GBPN containing disease nodes of “Tumor Grade 1,” “Intracranial Cystic Lesion,” “Hemangioblastoma,” “Benign Neoplasm,” etc. was assigned the class label “Abnormal Brain Growths,” as the majority of its nodes are associated with abnormal growths in the brain.

### DDrug repurposing or repositioning candidate identification

#### High-influence node identification

We calculated the degree, closeness, betweenness, eigenvector, and PageRank centrality for each node within their respective mc_GBPN. Each centrality measure detects the amount of influence a given node has over the flow of information in the mc_GBPN. Specifically, the degree centrality of a node is the number of edges connected to it. [43] Closeness centrality measures the average distance between a node and all other nodes in its mc_GBPN. [44] Betweenness centrality of a node is the percentage of shortest paths between any other pair of nodes in the graph which include the given node. [45–46] Eigenvector centrality measures the transitive influence of nodes; edges originating from a node with a high eigenvector centrality score contribute more to the score of the node they target than edges originating from a node with a lower eigenvector centrality score. Thus, if a node has a high eigenvector centrality score, it is connected to many other nodes with high eigenvector centrality scores. [47] We used 100 iterations in our eigenvector centrality calculations [48] (though we note that after experimenting with values ranging from 50 to 200 iterations, number of iterations had a negligible impact on the calculation and particularly did not affect the order of nodes from highest to lowest eigenvector centrality score). Finally, PageRank centrality is a subtype of eigenvector centrality that uses indegree rather than total degree. [49] We used the default probability setting in Gephi of 0.85 and the default epsilon setting 0.001 in our PageRank centrality calculations. [50] Note that all centrality scores will be greater than zero, and that closeness, eigenvector, and PageRank centrality must all be within the range of zero to one. [43–45, 48–49] In general across all metrics, higher centrality scores indicate a node is connected to a greater number of other nodes and/or is more centrally located within the network.

**Drug repurposing or repositioning candidate identification.** We ranked the five most influential nodes for each top ranked mc_GBPN by the five aforementioned centrality measures. We manually reviewed and selected the most interesting nodes from prioritized mc_GBPN based on their influence as potential candidates for drug repurposing **or repositioning** for GBM.

## Results

### Results of the GBPN

The NGKG contains 3,819,623 nodes and 84,223,681 edges from forty-three different biomedical data resources. Of these, 4,789 nodes and 177,106 edges were extracted and applied to generate the GBPN. After optimization, the GBPN contained 1,466 nodes (538 of which contained the merged information of two or more pre-optimization nodes) and 107,423 edges with average degree 73.276, defined as the total number of edges divided by the total number of nodes. Additional network properties can be found in Table 1.

**Table 1:** Network properties of the GBPN.

Network Property	Results
Nodes	1,466
Edges	107,423
Average Degree	73.276
Net Diameter ^a^	10
Average Path Length ^b^	3.751
Graph Density ^c^	0.05

^a^ The net diameter is defined as the maximal distance between all pairs of nodes. [51] ^b^ The average path length is defined as the average distance between all pairs of nodes. [52] ^c^The graph density is a measure of how close the graph is to being complete, and a complete graph contains all possible edges between any two nodes and has a density of 1. [53]

### Results of the mc_GBPN

We performed community detection by Louvain modularity [36] on the GBPN, obtaining forty-one mc_GBPN. Brief descriptions including class labels, number of nodes/edges and modularity scores for the ten mc_GBPN with the highest modularity scores are in Table 2. A full list of forty-one mc_GBPN is in the supplemental materials.

**Table 2:** Descriptions of the ten ranked mc_GBPN with the highest modularity score

mc_GBPN Index	Class labels	Nodes	Edges	Modularity Score
0	White Matter-Related Conditions	140	14,176	0.100
12	Movement Disorders	209	12,519	0.083
38	Seizures and Epilepsies	132	7,390	0.060
27	Amyotrophic Lateral Sclerosis and Related Conditions	156	5,984	0.052
21	Parkinsonism, Progressive Supranuclear Palsy, Dementia, and Related Conditions	94	5,936	0.048
14	Sensory and Motor Conditions	58	4,887	0.039
22	Neurodegeneration with Brain Iron Accumulation and Dystonias	65	3,904	0.033
16	Developmental Disorders	61	3,430	0.031
2	Dystonias and Mental Health Conditions	52	2,920	0.025
17	Cerebrovascular Conditions	75	2,735	0.024

**Fig. 4:**
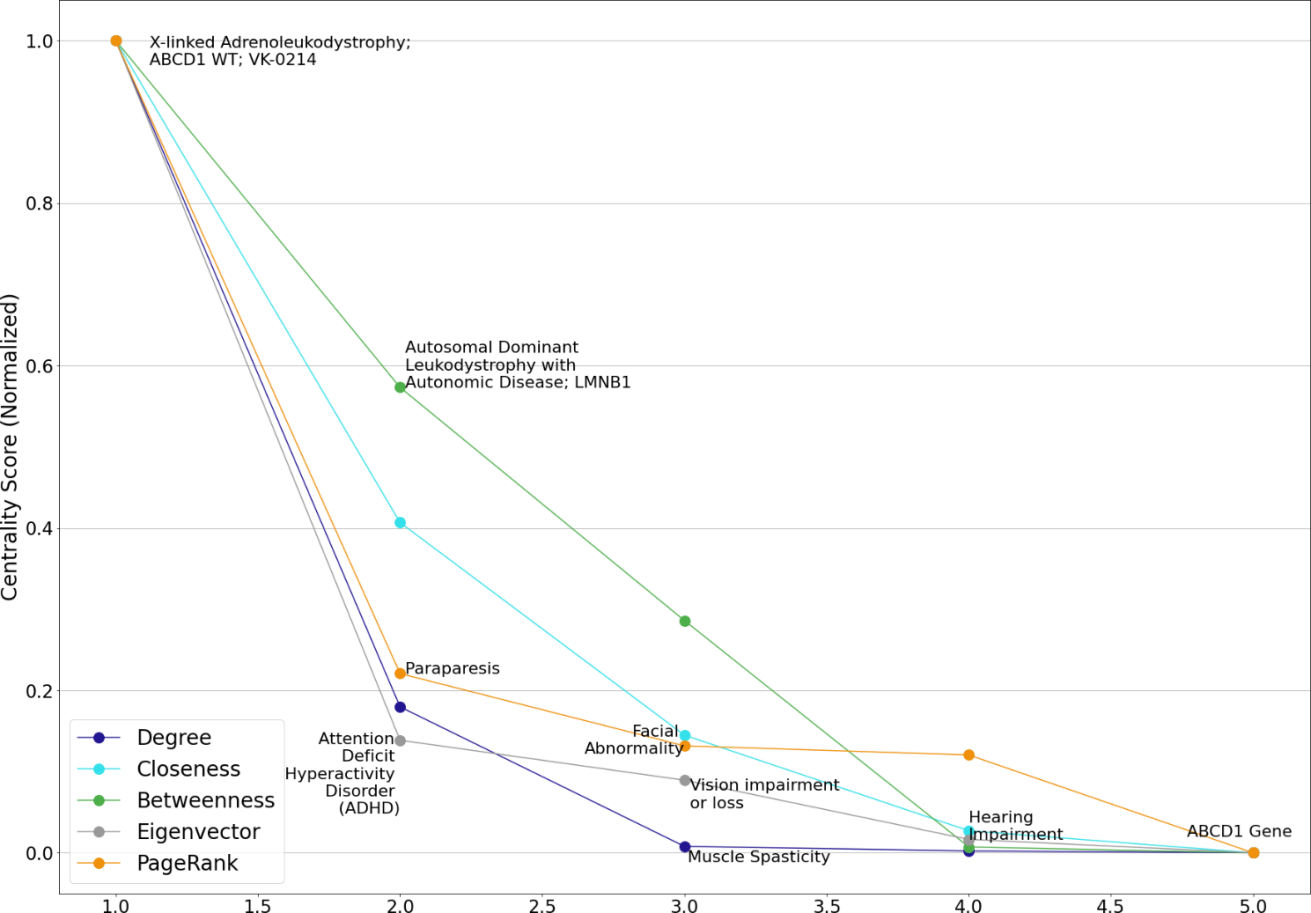
High-influence nodes identified by degree, closeness, betweenness, eigenvector, and PageRank centrality in mc_GBPN with an index of 0. The nodes displayed have a strong relationship to white matter-related conditions (as does GBM). Note that several nodes have high centrality scores across multiple measures; these nodes have a higher potential for drug repurposing or repositioning

We identified the five most influential nodes from each of the ten mc_GBPN (Table 2) by each centrality measure. The identified high-influence nodes from the mc_GBPN with an index of 0 are shown in Fig. 4. Centrality scores were normalized to a 0–1 range using the scikit-learn MinMaxScalar preprocessing function fit_transform method. [54] The full list of the five most influential nodes by each centrality measure within these ten mc_GBPN is in the supplemental materials.

### Drug repurposing or repositoning candidate identification

We examined the five most influential nodes from the top ten mc_GBPN (Table 2) by their centrality scores as potential candidates for drug repurposing or repositioning for GBM. We first normalized the centrality scores of the top five nodes by each centrality measure to a 0–1 range using the fit_transform method of the scikit-learn MinMaxScalar preprocessing function. [54] We then calculated a total normalized centrality score (TNCS) for each distinct node. The TCNS of a node is defined as the sum of its normalized centrality scores across degree, closeness, betweenness, eigenvector, and PageRank centralities. The TNCS of a node may range from 0 to 5, as there are five centrality measures. The nodes with the highest TCNS in each mc_GBPN listed in Table 2 are identified in Table 3. Of the nodes in Table 3, six had the highest centrality scores across all five centrality measures within their respective mc_GBPN.

**Table 3:** The most influential nodes in the mc_GBPN, selected by their TNCS across all centrality measures

Mc_GBPN Index	Node Description	TNCS
0	• X-linked Adrenoleukodystrophy (X-ALD) • ABCD1 WT Allele, ABC42, AMN, ACOX1, ECK2921, ECK1408 genes • VK-0214	4.407
12	• Ataxias, Choreas • SETX, WASF1, GCH1 genes	5
38	• Absence Epilepsy • CSTB, GABRA1, EC1 genes	3.986
27	• Amyotrophic Lateral Sclerosis (ALS) • ALS1, ALS2, IGFALS genes • Riluzole, Cannabidiol, Stem Cell Therapy	5
21	• Parkinson’s Disease • FBXO7, DCTN1, GBA, PARK1, PARK2, PARK5, PARK6, PARK7, PARK8 genes	4.413
14	• Spastic Paraplegia 10 (SPG10) • KIF5A gene	3.625
22	• Neurodegeneration with Brain Iron Accumulation 3 (NBIA3) • FTL gene	5
16	• Rett Syndrome • MECP2 gene	5
2	• Myoclonus Dystonia (M-D) • SGCE gene	5
17	• CADASIL and CARASIL Syndromes • NOTCH3, COL4A1, HTRA1 genes • Cerebrolysin, Palm Tocotrienol Complex	5

We observed that the most influential nodes in the mc_GBPN are associated with central nervous system conditions, [42] the main disease category GBM belongs to. Many are also genetic disorders and x-linked (e.g. x-linked adrenoleukodystrophy, Rett syndrome, [55] and some forms of Parkinson’s disease, [56] amyotrophic lateral sclerosis, [57] chorea, [58] and ataxia). [59].

The high-influence nodes in Table 3 shed light on drug repurposing or repositioning. For instance, a novel COL4A1 gene variant associated with CADASIL syndrome was recently found to be associated with GBM. [60] Moreover, the NOTCH3 gene (also associated with CADASIL syndrome) is a prognostic factor that promotes glioma cell proliferation, migration, and invasion. [61] Several drugs were identified as potential candidates for GBM, although they have not been clinically administered for GBM. Riluzole, a treatment for amyotrophic lateral sclerosis (ALS), has been shown to be an effective pretreatment that sensitizes glioma to radiation therapy. It also has synergistic effects in combination with select other drugs when used to treat GBM. [62] Cannabidiol, another ALS treatment, sensitizes GBM to TMZ in multiple orthotopic tumor models. [63] Inhalant cannabidiol has also been shown to inhibit the progression of GBM through regulation of the tumor environment. [64] Finally, stem cell therapy has shown potential for treating neuron and glial cell damage in the brain or spinal cord that results from neurological conditions such as GBM. [65] Interestingly, VK-0214 is currently being tested in a clinical trial as a treatment for x-linked adrenoleukodystrophy. [66] VK-0214 is a thyroid beta receptor agonist [67] which induces the ABCD2 gene by binding to and activating the thyroid beta receptor. [68] In *ABCD1* knockout mice, overexpression of ABCD2 via thyroid receptor activation has been shown to decrease the accumulation of very long chain fatty acids (VLCFA). [68] Based on these findings, selective thyroid receptor agonists are being evaluated as a novel treatment for X-ALD, which is characterized by the accumulation of VLCFA. [68] However, inhibition of fatty acid accumulation and oxidation has been shown to reduce GBM proliferation, [69] growth, [70] and survival [71] as well. The fatty acid accumulation-inhibiting effect of VK-0214 may be beneficial in the treatment of GBM. We will perform additional experimental validation as a next step. The full list of associations we examined between the nodes in Table 3 and GBM is in the supplemental materials.

## Discussion

In this study, we introduced an integrative GBM-based Biomedical Profile Network (GBPN) by integrating heterogeneous types of data, including disease, gene, drug, etc. based on their shared concept characteristics. To further construct focused subgraphs from the GBPN for supporting high-influence node identification for drug repurposing or repositioning, we derived modularity class-based subnetworks (mc_GBPN) by leveraging community detection, a form of graph clustering algorithm. Through implementing multiple network analysis techniques over the mc_GBPN, we successfully identified multiple high-influence nodes as potential drug repurposing candidates for GBM, as well as a candidate (VK-0214) for drug repositioning. This presented framework sheds light on supporting drug repurposing or repositioning in a more effective manner. While integrating more data to expand the search space, we organized the data in a more manageable scale with consideration of their relevance from the network view.

### Observations and findings

We applied a rare disease cluster consisting of 92 GBM-related diseases to construct the GBPN by exploring data from the NGKG. We optimized the GBPN for integrative rare disease profile generation by merging associated diseases, genes, treatments, etc. into singular nodes based on their shared concept names or external references. This approach allowed us to explore a large scale of GBM-relevant data in a concentrated and scalable form, which effectively supports drug repurposing or repositioning with lower computational burden as demonstrated in the Results section. As shown in Fig. 3, some level of inference was introduced during the optimization. When we merged Lafora disease, EPM2A, EPM2B and Metformin, we declared the new connections between Metformin and EPM2A and EPM2B based on inference, since there are no existing connections among them obtained from the NGKG. Since PME2 shares different degrees of associations (different numbers of edges) with EPM2A, EPM2B and Metformin, we inferred these four concepts are potentially associated with each other, leading to node merging. The findings from Bisulli et al. [72] proved the inference introduced for this particular case. In the future study, we will attach relevant references gathered from the previously developed scientific annotation knowledge graph, [73] to the merged nodes, as scientific evidence enrichment.

After GBPN optimization, we generated focused subgraphs of the GBPN by performing community detection as a graph clustering algorithm, resulting in a network partitioned into modularity classes (mc_GBPN). mc_GBPN as a set of subgraphs (i.e., clusters) derived from the GBPN were ranked by their modularity scores, which allowed programmatically upgrade those top prioritized clusters for further investigation and downgrade those with lower priority. Our experiments showed that such a strategy did not lose any important information compared to the GBPN, instead more high-influence nodes were exposed in the top ranked clusters for easy extraction. For instance, nine distinct top high-influence nodes derived from the GBPN appear in the top five most influential node lists from their respective mc_GBPN. We calculated the five most influential nodes by each centrality measure in the GBPN and found that seven of the ten most-influential nodes (see Table 4) were included in the resulting list. The remaining nodes (i.e., Spastic Paraplegia 10, Rett syndrome, Myoclonus Dystonia) were present exclusively in the lists of high-influence nodes derived from the mc_GBPN. The complete lists of the five most influential nodes by each centrality measure in the GBPN and in each modularity class of the mc_GBPN are in the supplemental materials.

**Table 4:** The top five nodes in mc_GPBN #0 ranked by five centralities

	Degree	Closeness	Betweenness	Eigenvector	PageRank
1	X-linked Adreno- leukodystrophy; ABCD1 WT; VK-0214	Abnormal Eye	X-linked Adreno- leukodystrophy; ABCD1 WT; VK-0214	X-linked Adreno- leukodystrophy; ABCD1 WT; VK-0214	X-linked Adreno- leukodystrophy; ABCD1 WT; VK-0214
2	Autosomal Dominant Leukodystrophy with Autonomic Disease; LMNB1	X-linked Adreno- leukodystrophy; ABCD1 WT; VK-0214	Autosomal Dominant Leukodystrophy with Autonomic Disease; LMNB1	Attention Deficit Hyperactivity Disorder (ADHD)	Paraparesis
3	Muscle Spasticity	Autosomal Dominant Leukodystrophy with Autonomic Disease; LMNB1	Paraparesis	Vision impairment or loss	Facial Abnormality
4	Paraparesis	Progressive Disorder	Muscle Spasticity	Hearing Impairment	Autosomal Dominant Leukodystrophy with Autonomic Disease; LMNB1
5	Cognitive Decline/ Impairment	Muscle Spasticity	Cerebro-medullospinal Disconnection	Dementias, Amentia	ABCD1 Gene

### Limitations of this study

Due to lack of standardization across the biomedical resources, integrating information from different resources with a high level of precision proved to be a significant challenge. While we optimized the GBPN by merging nodes with closely associated information into a singular node, we were not able to fully automate this process because the data was not represented in a standard form and the nature of the NGKG that does not contain predefined data models, instead a rule-based semi-automatic approach. A more sophisticated harmonization process will be proposed when we obtain data to build the GBPN. For instance, rare diseases from different resources will be harmonized and standardized by using GARD ID, genes with HGNC ID, etc. Additionally, during the step of high-influence node identification, we manually searched for scientific evidence to support our findings. In the future study, we will programmatically query the rare disease-based scientific annotation knowledge graph [73] for evidence collection. In the future study, we will adopt/extend the strategy of network optimization to apply on the datasets with well-defined data models underneath, then we will be able to generate highly condensed graphs by merging nodes/relationships by different concept types.

As a feasibility study, our aim is to prove the capability of our presented computational approach for supporting drug repurposing or repositioning. The findings were examined with published scientific evidence. For instance, we identified indirect evidence discussed in the section of Drug Repurposing Candidate Identification, to support that the fatty acid accumulation inhibiting effect of VK-0214 may be beneficial in the treatment of GBM. Experimental validation is out of scope of this study, which is planned as a next step.

### Future directions

We presented a preliminary analysis of GBM-related data that allowed us to identify potential candidates for drug repurposing and repositioning to treat the condition. Although scientific evidence has been identified to support our initial findings, experimental validation is necessary to determine whether these candidates would be effective in treating GBM patients in practice. Clinical observations/efficacy regarding those candidates administered for patients with GBM, derived from Electronic Medical Records (EMR) can serve as another layer of validation. We propose to mine clinical data from National COVID Cohort Collaborative (N3C) and the Biomedical Translational Research Information System (BTRIS) at NIH for clinical evidence identification. Our pipeline is modularized as shown in Fig. 1, thus we propose to extend the use of each module. We will expand to other disease areas by starting with other disease clusters and generating corresponding GBPN. We also propose to explore other clustering algorithms besides Louvain community detection for focused subgraph generation (e.g. Leiden [74] community detection), as each algorithm will have different conditions for what defines a cluster and may therefore unearth different candidate nodes (or return a more refined list). Besides the application of drug repurposing we started with, we believe mc_GBPN as a collection of rare disease profiles providing a complete picture of direct and indirect associations to the target disease can be a valuable source to help us understand the etiology of rare diseases.

## Conclusion

In this study we presented a preliminary network analysis-based approach to drug repurposing and repositioning for GBM. We successfully identified several potential candidates.

via centrality and community detection calculations, and substantiated the connections between these candidates and GBM. We reinforced the findings of emerging studies into some treatments and also identified a new candidate, VK-0214, that could be potentially repurposed to treat GBM. These findings can guide future experimental validation, which could lead to new, more effective treatments that extend the lifespan of patients living with GBM.

### Electronic supplementary material

Below is the link to the electronic supplementary material.

Supplementary Material 1

Supplementary Material 2

Supplementary Material 3

Supplementary Material 4

## Data Availability

The full documentation, codes, and other supplemental data is found on GitHub (https://github.com/ncats/drug_rep/tree/main/Glioblastoma_Subgraph ).
