# Supplementary material for: Integrative rare disease biomedical profile based network supporting drug repurposing or repositioning, a case study of glioblastoma
Source: Orphanet J Rare Dis. 2023 Sep 25;18:301. doi: 10.1186/s13023-023-02876-2 (PMC10519087; doi:10.1186/s13023-023-02876-2)
Supplement: Supplementary file 1 — Supplementary Material 1 [file 13023_2023_2876_MOESM1_ESM.pdf]

We see that Gephi divides the network into 41 modularity classes (labeled with numbers 0-40). These modularity classes have a range of sizes, with the largest being comprised of 14.26% of the total nodes (209 nodes out of 1466) and the smallest being comprised of only 0.07% (1 node out of 1466). The full list of which modularity class each node belongs to can be found here. Glioblastoma itself is in modularity class 4.

0. **Modularity Class 0** (140 nodes): White Matter-Related Conditions
1. **Modularity Class 1** (18 nodes): Focal Sensory Auditory Seizures and Related Conditions
2. **Modularity Class 2** (52 nodes): Dystonias and Mental Health Conditions
3. **Modularity Class 3** (75 nodes): Nervous System Conditions
4. **Modularity Class 4** (2 nodes): Glioblastoma
5. **Modularity Class 5** (28 nodes): Speech and Language Conditions
6. **Modularity Class 6** (58 nodes): Alzheimer's Disease and Related Conditions
7. **Modularity Class 7** (2 nodes): Mosquito-borne Viral Encephalitis
8. **Modularity Class 8** (1 node): Marchiafava-Bignami disease
9. **Modularity Class 9** (1 node): Post-Polio Syndrome
10. **Modularity Class 10** (1 node): X-Linked Conditions
11. **Modularity Class 11** (1 node): Botulism
12. **Modularity Class 12** (209 nodes): Movement Disorders
13. **Modularity Class 13** (1 node): Trichinosis
14. **Modularity Class 14** (58 nodes): Sensory and Motor Conditions
15. **Modularity Class 15** (20 nodes): Gastrointestinal Tract-Related Conditions
16. **Modularity Class 16** (61 nodes): Developmental Disorders
17. **Modularity Class 17** (75 nodes): Cerebrovascular Conditions
18. **Modularity Class 18** (1 node): Idiopathic Hypersomnia
19. **Modularity Class 19** (81 nodes): Encephalopathies and Associated Conditions
20. **Modularity Class 20** (1 node): Paroxysmal Hemicrania
21. **Modularity Class 21** (94 nodes): Parkinsonism, Progressive Supranuclear Palsy, Dementia, and Related Conditions
22. **Modularity Class 22** (65 nodes): Neurodegeneration with Brain Iron Accumulation and Dystonias
23. **Modularity Class 23** (1 node): Burning Mouth Syndrome
24. **Modularity Class 24** (27 nodes): Tumors
25. **Modularity Class 25** (1 node): New-Onset Refractory Status Epilepticus
26. **Modularity Class 26** (1 node): Neurocysticercosis
27. **Modularity Class 27** (156 nodes): Amyotrophic Lateral Sclerosis and Related Conditions
28. **Modularity Class 28** (1 node): Subependymal Nodular Heterotopia
29. **Modularity Class 29** (1 node): Amoebiasis Due to Free-Living Amoebae
30. **Modularity Class 30** (2 nodes): Acute Disseminated Encephalitis and Encephalomyelitis
31. **Modularity Class 31** (71 nodes): Prion Diseases
32. **Modularity Class 32** (3 nodes): Viral Infections and Zoonotic Diseases
33. **Modularity Class 33** (1 node): Opsoclonus-Myoclonus Syndrome

34. **Modularity Class 34** (1 node): Disembarkment Syndrome
35. **Modularity Class 35** (1 node): Progressive Multifocal Leukoencephalopathy
36. **Modularity Class 36** (3 nodes): Childhood Developmental and Mental Disorders
37. **Modularity Class 37** (3 nodes): Parasitic Infections
38. **Modularity Class 38** (132 nodes): Seizures and Epilepsies
39. **Modularity Class 39** (1 node): Reversible Cerebral Vasoconstriction Syndrome
40. **Modularity Class 40** (15 nodes): Corpus Callosum Conditions
