# Supplementary material for: Integrative rare disease biomedical profile based network supporting drug repurposing or repositioning, a case study of glioblastoma
Source: Orphanet J Rare Dis. 2023 Sep 25;18:301. doi: 10.1186/s13023-023-02876-2 (PMC10519087; doi:10.1186/s13023-023-02876-2)
Supplement: Supplementary file 4 — Supplementary Material 4 [file 13023_2023_2876_MOESM4_ESM.docx]

**NGKG Resources**

| Genetic and Rare Diseases Information Center (GARD) |
| --- |
| Genetics Home Reference (GHR) |
| National Organization for Rare Disorders (NORD) |
| Disease Ontology |
| Human Phenotype Ontology |
| Orphanet in Spanish |
| Medical Subject Headings (MeSH) |
| MEDLINEPLUS |
| Online Mendelian Inheritance in Man (OMIM) |
| ICD10CM |
| Orphanet_prevalence.xml |
| Orphanet |
| NCI Thesaurus |
| HPO Annotation |
| Veterans Health Administration National Drug File (VANDF) |
| CLINVAR |
| Cell Line Ontology |
| BRENDA Tissue and Enzyme Source Ontology |
| Drug Database for Inborn Errors of Metabolism (DDIEM) Ontology |
| Cell Ontology |
| Uber-anatomy ontology |
| Gene Ontology |
| Genotype Ontology |
| Ontology of Genes and Genomes |
| Pathway Ontology |
| Mammalian Phenotype Ontology |
| Ontology of Adverse Events |
| Name Reaction Ontology |
| Phenotypic Quality Ontology |
| Foundational Model of Anatomy |
| Chemical Entities of Biological Interest (ChEBI) |
| Inxight Drug |
| FDA Orphan Drug Designations |
| Orphanet_NaturalHistoryStudy |
| Orphanet_HPOannotations |
| Orphanet_GeneAssociations |
| MEDGEN |
| GENEREVIEWS |
| The Experimental Factor Ontology (EFO) |
| Mondo Disease Ontology (MONDO) |
| Orphanet_DiseaseMappings |
| NEWBORN Screening |
| Monogenic Disease |
